# Radioresistant cervical cancer shows upregulation of the NHEJ proteins DNA-PKcs, Ku70 and Ku86

**DOI:** 10.1038/sj.bjc.6605201

**Published:** 2009-08-11

**Authors:** C Beskow, J Skikuniene, Å Holgersson, B Nilsson, R Lewensohn, L Kanter, K Viktorsson

**Affiliations:** 1 Department of Gynaecologic Oncology, Radiumhemmet, Karolinska University Hospital, Solna, Stockholm SE-171 76, Sweden; 2 Department of Pathology and Cytology, Karolinska University Hospital, Solna, Stockholm SE-171 76, Sweden; 3 Department of Oncology and Pathology, Karolinska Institutet, Solna, Stockholm SE-171 76, Sweden; 4 Department of Oncology and Pathology, Karolinska Biomics Center, Karolinska Institutet, Solna, Stockholm SE-171 76, Sweden

**Keywords:** cervical cancer, radioresistance, DNA-PK, p53, p21

## Abstract

**Background:**

Radiotherapy is central in the treatment of cervical cancer. The formation of DNA double-strand breaks is considered to be critical for the radiotherapeutic effect. The non-homologous end joining (NHEJ) proteins DNA–PKcs, Ku70 and Ku86 have a major role in repairing DNA lesions. The objective of this study was to analyse if the expression of DNA–PKcs, Ku70 and Ku86 and their downstream signalling molecules p53, p21 and Mdm-2 are altered in residual cervical tumours after radiotherapy.

**Methods:**

Retrospective analysis of 127 patients with cervical cancer stage IB-IIA treated with preoperative radiotherapy and radical surgery, revealed residual tumour in the cervical specimen in 30 patients. In 22 cases tumour material from residual and corresponding primary tumour were retrieved and the expression of DNA–PKcs, Ku86, Ku70, p53, p21 and Mdm-2 were assessed by immunohistochemistry.

**Results:**

Residual tumours showed increased frequency of DNA–PKcs (*P*=0.037), Ku70 (*P*=0.018), Ku86 (*P*=0.008) positive cells. A correlation in DNA–PKcs expression between primary and residual tumours was found. The frequency of p21-positive cells was decreased (*P*=0.007) in residual tumours whereas no change in p53 or Mdm-2-positive cells were observed.

**Conclusion:**

Our results show that cervical carcinoma surviving radiotherapy have an increased DNA–PK expression. Studies on larger patient cohorts are needed to allow an interpretation that an upregulation of DNA–PK function may be part of a radioresistance mechanism within this tumour type.

Radiotherapy (RT) is an established treatment modality for cervical carcinoma. There are, however, cases not responding properly to such treatment and an increased understanding of the underlaying molecular processes leading to radioresistance may lead to novel radiosensitising strategies.

Analysis of markers in cervical tumour specimens before RT have shown that hypoxia (Fyles *et al*, 1998; Haensgen *et al*, 2001) as well as increased survival signalling through Her-2 (Lee *et al*, 2005), epidermal growth factor receptor (EGFR) or insulin-like growth factor receptor (IGFR) (Kim *et al*, 2006) are predictive for lack of RT response.

The most critical DNA lesion induced by radiation is DNA double-strand breaks (DNA dsbs) and in mammalian cells these lesions are predominantly repaired by non-homologous end joining (NHEJ) (Hefferin and Tomkinson, 2005). Central in the NHEJ process is the binding of Ku70/Ku86 proteins to the free DNA ends followed by recruitment of the catalytic subunit of DNA-dependent protein kinase (DNA-PKcs), which in a phosphorylation cascade regulate proteins operative in the DNA dsbs ligation process (Hefferin and Tomkinson, 2005). The importance of DNA-PK in radiation responses are illustrated by studies on the human glioblastoma cell lines M059K and M059J, proficient and deficient in DNA-PKcs, which show large differences in radioresponsiveness with the latter being hypersensitive (Allalunis-Turner *et al*, 1995; Lees-Miller *et al*, 1995).

Apart from a critical role in DNA repair activated DNA-PKcs also phosphorylate proteins involved in control of cell cycle checkpoints and different cell-death pathways, the tumour suppressor p53 being one example (Kastan *et al*, 1991; Lees-Miller *et al*, 1992). Upon such phosphorylation p53 is stabilised and may cause increased expression in the cell cycle regulator p21 or the proapoptotic protein Bax (Bernstein *et al*, 2002).

The level of p53 is controlled by the human homologue to the mouse double minute 2 protein (Mdm-2), which in a ubiquitin-dependent manner targets p53 for degradation through the proteasome (Haupt *et al*, 1997; Kubbutat *et al*, 1997). However, in the majority of cervical cancers inactivation of p53 is mainly caused by human papillomavirus infection (Scheffner *et al*, 1990).

Given the prominent role of DNA-PK in DNA-dsb repair, we and others have examined if expression of DNA-PKcs or Ku proteins in primary cervical tumour biopsies can predict an RT response (Wilson *et al*, 2000; Beskow *et al*, 2006). Using immunohistochemical analysis of Ku70 and Ku80 on primary tumour biopsies from stage I–III cervical cancer patients, Wilson *et al* (2000) showed that all tumours with a low frequency of Ku70 or Ku80 immunopositive cells (<60%) were radiosensitive in a clonogenic assay. We previously reported that the response to preoperative intracavitary RT measured as pathologic complete remission in the surgical specimen derived from patients with cervical cancer stage IB and IIA could not be predicted by the expression of DNA-PK proteins in primary tumour biopsies (Beskow *et al*, 2006).

Few clinical studies have so far addressed the question of how radiotherapy may influence the expression of the DNA-PK complex by analysing primary and residual tumour tissue after radiotherapy. One may hypothesize that radioresistant tumours would display increased function of a DNA-PK and p53-signalling network. In this study, we aimed to compare the expression of DNA-PK proteins, p53, p21 and Mdm-2 in primary tumours and corresponding residual cervical tumours after RT using immunohistochemistry.

## Materials and methods

### Patients

This report on 22 patients is based on a retrospective analysis of 127 patients with cervical cancer FIGO stage IB-IIA consecutively admitted to Karolinska University Hospital during January 1989–December 1991 (Beskow *et al*, 2002). Treatment included preoperative RT followed by radical surgery, which was performed 4 weeks after completed RT. In 22 out of 30 cases with residual cervical tumours it was possible to retrieve tumour tissue from the corresponding primary tumour. Clinical and pathological characteristics of these 22 cases are shown in Table 1. The use of patient materials was approved by the Ethical Committee (Ethical permit; Dnr 00-343) at the Karolinska Institutet and informed consent was obtained from all patients alive at the time of analysis.

### Radiotherapy

Preoperative RT was given as brachytherapy to 18 patients, whereas one patient received external beam radiation therapy and three patients received a combination of external beam radiation treatment and one or two fractions of brachytherapy. The treatment given to each patient is described in Table 1. Brachytherapy was applied according to ‘the modified Stockholm method’ using two uterovaginal insertions with a 3-week interval (Beskow *et al*, 2002). During the period 1989–1991 our technique for brachytherapy gradually changed from a manual technique using radium to a remote after-loading technique using cesium-137 in the Selectron system. When introducing the after-loading technique, the dose prescriptions were calculated to mimic the doses given by the manual technique. Ten patients were treated with radium applicators with a dosage quoted in milligram-hours of radium (mghRa) calculated to a mean fraction dose of 3300 mghRa and a mean dose rate of 1.1 Gy h^−1^ to point A (ICRU, 1985). Eleven patients received brachytherapy with the remote after-loading technique using cesium-137 with a mean fraction dose of 22 Gy and a mean dose rate of 1.35 Gy h^−1^ to point A. Treatment with external beam irradiation over the pelvis was given by linear accelerators (6–21 MV) to four patients with a daily fraction of 1.6 Gy given 5 days per week. The prescribed doses varied between 13 and 30 Gy (Table 1).

### Immunohistochemistry

Expression of DNA-PKcs, Ku86, Ku70, Mdm-2, p53 and p21 proteins were assessed on formalin-fixed tumour biopsies taken before RT and from the corresponding formalin-fixed surgical specimens. Immunostainings were done using the avidin–biotin–peroxidase complex- (ABC) technique according to the manufacturer's instructions (Elite Standard Kit., Vector Laboratories, Burlingame, CA, USA) and as previously described (Beskow *et al*, 2006). Briefly, paraffin sections (4 *μ*m) were dewaxed and rehydrated. The slides were boiled in citrate buffer (pH 6.0, 12 min at 800 W and 20 min at 250 W), cooled and rinsed with phosphate-buffered saline. After blocking with 1% BSA, the following primary mouse monoclonal antibodies were used; DNA-PKcs, Ku86, Ku70 (Neo Markers, Fremont, CA, USA), p53 (clone DO-7, DAKO), p21 (clone EA10, Oncogene Research Products, Cambridge, MA, USA) and Mdm-2 (SMP14, Santa Cruz Biotechnology, Santa Cruz, CA, USA). The staining was visualised using a biotinylated secondary antibody followed by ABC and diaminobenzedine as a chromogene (Vector Laboratories) and slides counterstained with haematoxylin. Evaluation of staining was carried out by a trained pathologist in a blinded fashion and in each specimen 500 tumour cells were counted. The percentage of stained cells was assessed and the intensity graded as low, intermediate or strong. Morphologically non-neoplastic cells in each specimen served as internal negative controls for the staining procedure.

### Statistical analysis

Comparison between primary biopsies and residual tumours were determined using paired samples *t*-test and Wilcoxon's Signed Ranks test and the relations between primary and residual tumour variables were evaluated with Spearman's correlation test.

## Results

### Higher frequency of DNA-PKcs, Ku70 and Ku86 immunopositive cells in residual cervical carcinoma specimen

**Figure 1:**
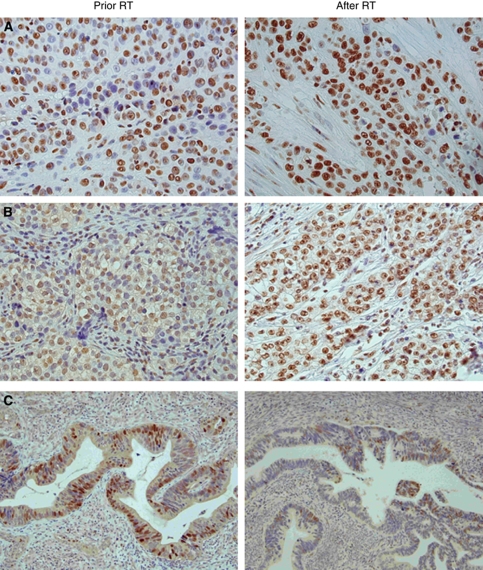
(**A**–**C**) Positive immunohistochemical staining of Ku70 (**A**) in poorly differentiated squamous cell carcinoma, Ku86 (**B**) in glassy cell adenocarcinoma and p21 (**C**) in moderately differentiated adenocarcinoma of the cervix. Immunoreactivity is noted in nuclei of the tumour cells. After radiotherapy an increased frequency of Ku70- and Ku86-positive tumour cells are seen parallel to a decrease in p21 positivity.

The percentages of cells staining positive for DNA-PKcs, Ku86 and Ku70 in primary biopsies and residual tumours for each patient are listed in Table 2. Residual tumours were found to display higher percentages of DNA-PKcs immunopositive cells (Mean±s.e.m.: 67.8±5.5), compared with the corresponding primary tumour biopsy (Mean±s.e.m.: 57.8±5.2) (mean diff=9.95, s.d.=20.4, *P*=0.037). Similarly, the frequency of cells staining positive for Ku70 and Ku86 were higher in residual than in primary tumours. Expression of Ku70 in primary tumours ranged from 48 to 95% (Mean±s.e.m.: 76.9±3.6) and from 51 to 98% (Mean±s.e.m.: 86.8±3.2) in residual tumours after RT (mean diff=9.91, s.d.=17.6, *P*=0.018) (Figure 1A). The expression of Ku86 varied between 0–98% (Mean±s.e.m. 66.7±5.4) in primary tumours, whereas in residual tumours the percentage of positive cells ranged from 55 to 100% (Mean±s.e.m.: 83.3±3.0) (mean diff=16.7, s.d.=26.1, *P*=0.008) (Figure 1B). Corresponding analysis yielded *P*-values of 0.029, 0.006 and 0.004, respectively. A correlation was observed between DNA-PKcs in primary and residual tumours (rho=0.55, *P*=0.010) (data not shown). In contrast, no correlations were found between primary and residual tumours for neither Ku70 (rho=0.126, *P*=0.586) nor Ku 86 (rho=0.323, *P*=0.154) proteins. All three proteins displayed a predominantly nuclear localisation. No overall differences in staining intensity could be detected when comparing primary and corresponding residual tumours for either DNA-PKcs or the Ku proteins. As seen in Table 2, there is a variation in positivity for the DNA-PK components among both the primary and residual tumour tissue. However, these differences in immunopositivity were not found to correlate with either clinical characteristics, that is, tumour size, histopathologic type or grade or with the RT regimen given.

### Decreased frequency of p21 staining in residual cervical carcinoma specimen

The frequency of tumour cells positive for p53, p21 or Mdm-2 in tumour biopsies taken before therapy and in residual tumours is presented in Table 3. The percentage of p21-positive cells was decreased in residual tumours (Mean±s.e.m.: 9.35±3.4%) compared with corresponding primary tumours (Mean±s.e.m.: 24.6±4.4%) (mean diff=−15.2, s.d.=22.4, *P*=0.007). Corresponding analysis gave a *P*-value of 0.010 (Figure 1C). In contrast, no significant difference in the frequency of p53-stained tumour cells could be detected when primary tumour biopsies (Mean±s.e.m.: 7.36±2.2%) and residual tumours were compared (Mean±s.e.m.: 7.50±4.4%) (mean diff=0.14, s.d.=14.2, *P*=0.965). The Mdm-2 positivity of the cervical tumour cells ranged from 0 to 100%, with no overall difference in frequency between specimens taken before (Mean±s.e.m.: 65.3±6.8%) and after RT (Mean±s.e.m.: 65.6±9.4%) (mean diff=0.32, s.d.=39.8, *P*=0.970). Staining of p53 and p21 revealed a predominant nuclear localisation, whereas Mdm-2 displayed a cytoplasmatic staining pattern in 50% of the immunopositive primary tumours. The majority of these cases also showed cytoplasmatic staining in the corresponding residual tumours. No overall difference in intensity of immunostaining was found when comparing primary and residual tumours for p21, p53 or Mdm-2.

## Discussion

Impaired RT responsiveness of tumours is a major clinical problem in several solid tumour types including cervical carcinoma, in which RT is one of the key treatment modalities used. Activation of DNA damage and DNA repair networks are central in the molecular responses to radiation and within these networks the DNA-PK complex have a major function. The results of this study, comparing the expression of DNA-PK proteins in primary tumour biopsies and corresponding residual cervical carcinoma after RT, show a significant increase in frequency of DNA-PKcs, Ku70 and Ku86-positive cells in the residual tumours.

To our knowledge this is the first study of clinical cervical cancer in which DNA-PK complex expression has been examined in both primary and residual tumour tissue after RT. There is one study analysing DNA-PK proteins in oral squamous cell carcinoma tumours pre- and post RT (Shintani *et al*, 2003). In that study, patients were treated with external beam radiotherapy (EBRT) with a total mean dose of 32.9 Gy followed by surgery performed after 2–3 weeks. An increased expression of DNA-PKcs and Ku70 was observed in residual tumour, whereas the percentage of the Ku80-positive cells was similar in tumour tissue pre- and post RT. The results reported by Shintani *et al* (2003) are partly in accordance with ours, suggesting that upregulation of DNA-PKcs and Ku70 associates with radioresistance. However, the discrepancy with respect to Ku80 between the studies is not clearly understood but may be related to differences in treatment type, intracavitary *vs* EBRT, or to the effect of chemotherapy, which was given to half of the patients in the study of Shintani *et al* (2003) Moreover, differences in tumour type as well as in the time span between end of RT and surgery may contribute.

Several studies have been performed with the aim of identifying molecular markers that could predict RT response in cervical cancer (Fyles *et al*, 1998; Haensgen *et al*, 2001; Lee *et al*, 2005; Kim *et al*, 2006). Thus it has been shown that the downstream signalling component of EGFR, phosphorylated AKT, had a significantly higher expression in radioresistant cases compared with cancer tissue obtained from a radiosensitive group of cervical cancer patients in whom radiosensitivity was defined as no local recurrence at least 3 years after treatment (Kim *et al*, 2006). In preclinical studies of tumour cell lines, radiation has been shown to activate growth factor signalling networks, for example, EGFR and IGFR leading to inhibition of radiation-induced cell death (Dittmann *et al*, 2005a, 2005b; Fuhrman *et al*, 2008). Interestingly, recent data suggest that radiation-induced activation of EGFR may result in accumulation of EGFR within the cell nucleus, causing an increased function of the DNA-PK complex (Dittmann *et al*, 2005b). Moreover, in a cervical carcinoma model it was shown that addition of the PI3 kinase inhibitor, LY294002, sensitised tumour cells to radiation in part by inhibiting the function of DNA-PK (Fuhrman *et al*, 2008). In the context of these findings, one may hypothesise that the increased DNA-PK observed in residual cervical tumour tissue is a consequence of a radiation-induced increase in growth factor receptor signalling. Ongoing clinical studies in cervical cancer in which EGFR inhibition is combined with RT will evaluate if such treatment combination will increase radiosensitivity and if it involves inhibition of DNA-PK.

We also analysed the expression of p53, p21 and Mdm-2 proteins in cervical tumour tissue before and after RT. A statistically significant decrease in the frequency of p21 immunopositive cells was found in residual tumours after RT compared with corresponding primary tumour. In contrast, no difference in the frequency of p53 and Mdm-2 positive cells was observed between residual and primary tumours. Thus the downregulation of p21 seems to be independent of p53. Such p53-independent regulation has been previously described (Bernstein *et al*, 2002). In tumour cell line models, it is well established that a radiation-induced increase in p21 expression does occur (Bernstein *et al*, 2002); however, few studies have examined how radiation may influence p21 expression in a clinical setting. Yet, Niibe *et al* (1999) reported that the number of p21- and p53-positive cells increased after RT in tumour tissue taken before treatment and 6 h after the fifth dose of 1.8 Gy from patients with cervical cancer FIGO stage II-IVA. These results are in marked contrast to ours, in which residual cervical tumours showed a downregulation of p21 expression and may be explained by several factors. Thus, in the study of Niibe *et al* (1999) the biopsies were taken in the early phase of the radiation course, at a total dose of approximately 10 Gy, which is in contrast to the total dose of at least 30 Gy given in our study. Moreover, Niibe *et al* (1999) analysed p21 expression at 6 h post radiation, that is, at ongoing radiation-induced cell cycle perturbations, while our analysis was performed 4 weeks after end of RT. One potential mechanism that could explain the observed decrease in p21 expression in RT resistant tumours is that tumour cells with high p21 expression or with capacity to induce p21 expression will be arrested either at G1 or at G2/M, whereas tumour cells with low p21 expression will progress through the cell cycle after radiation and will thereby constitute a larger proportion of the residual tumour.

In conclusion, we report here that residual cervical tumours after RT show higher percentages of cells staining positive for DNA-PKcs, Ku70 and Ku86 compared with corresponding primary tumours. In contrast, we observe a decreased expression of the cell cycle-regulating protein p21 in residual tumours. Our results suggest that one mechanism of radioresistance in cervical cancer may include upregulation of the DNA-PK proteins. However, the findings reported in this study need to be confirmed in larger patient materials in order to draw the conclusion of a definite role of the DNA-PK proteins in radioresistance of cervical carcinoma.

## Figures and Tables

**Table 1:** Characteristics of 22 patients with residual tumour after radiotherapy

**Patient**	**Age**	**FIGO stage**	**Tumour size (cm)**	**Histopathological type**	**Histopathological grade**	**Preoperative radiotherapy**
1	32	IB	3.0	Squamous	Low	Brachy Cs^a^
2	43	IB	8.0	Squamous	Moderate	Brachy Ra^b^
3	43	IB	5.0	Squamous	Moderate	Brachy Ra
4	41	IB	4.5	Adenocarcinoma	High	Brachy Cs
5	39	IB	6.0	Squamous	Low	Brachy Cs
6	28	IB	8.0	Squamous	Low	Brachy Ra
7	74	IB	5.0	Squamous	High	Brachy Ra
8	44	IB	5.0	Squamous	High	Brachy Cs
9	43	IB	1.0	Adenocarcinoma	High	Brachy Cs
10	33	IB	4.5	Adenosquamous	Low	Brachy Ra
11	39	IB	4.0	Adenocarcinoma	Low	Brachy Cs
12	39	IB	5.0	Adenocarcinoma	Moderate	Brachy Cs
13	28	IB	5.5	Adenocarcinoma	Moderate	Brachy Cs
14	38	IIA	6.0	Squamous	Low	Brachy Cs
15	54	IIA	3.5	Adenocarcinoma	Moderate	Brachy Ra
16	25	IIA	6.0	Squamous	Low	Brachy Ra
17	52	IIA	7.0	Squamous	Low	Ext 30Gy^c^
18	79	IIA	5.0	Adenocarcinoma	High	2 Ra+20Gy^d^
19	71	IIA	4.0	Squamous	Moderate	Brachy Ra
20	45	IIA	4.0	Squamous	Moderate	Brachy Cs
21	25	IIA	5.0	Adenocarcinoma	Low	30Gy+1Ra^e^
22	43	IIA	6.0	Squamous	Moderate	2Cs+13Gy^f^

a Brachy Cs=two fractions of brachytherapy using Cesium.

b Brachy Ra=two fractions of brachytherapy using Radium.

c External beam radiation 30 Gy.

d Two fractions of brachytherapy using Radium and external radiation 20 Gy.

e External radiation 30 Gy and one fraction of brachytherapy using Radium.

f Two fractions of brachytherapy using Caesium and external radiation total dose of 13 Gy. Surgery was performed 3 months after end of radiotherapy.

**Table 2:** Percentage of cells staining for DNA-PKcs, Ku70 and Ku86 in primary and corresponding residual tumours after radiotherapy

	**DNA-PKcs**			**Ku70**			**Ku86**		
**Patient**	**Primary tumour %**	**Residual tumour %**	***d***	**Primary tumour %**	**Residual tumour %**	***d***	**Primary tumour %**	**Residual tumour %**	***d***
1	77	72	−5	72	84	12	78	83	5
2	83	100	17	73	98	25	87	91	4
3	84	75	−9	93	95	2	45	95	50
4	78	80	2	95	97	2	84	74	−10
5	50	86	36	72	96	24	41	94	53
6	60	88	28	91	96	5	0	95	95
7	94	90	−4	94	80	6	90	100	10
8	42	50	8	81	95	14	83	80	−3
9	78	71	−7	54	75	21	87	90	3
10	69	34	−35	89	98	9	98	98	0
11	25			91	90	−1	79	82	3
12	41	95	54	92	93	1	77	58	−19
13	51	49	−2	51	51	0	46	58	12
14	41	90	49	48	98	50	53	90	37
15	33	33	0	51	73	22	36	55	19
16	71	94	23	94	95	1	90	98	8
17	19	27	8	62	65	3	59	71	12
18	10	30	20	78			50		
19	73	83	10	65	98	33	80	84	4
20	81	91	10	91	55	−36	86	85	−1
21	28	31	3	79	97	18	52	92	40
22	51	54	3	76	93	17	49	77	28
Mean *d*			9.95			9.91			16.7
s.d.			20.4			17.6			26.1

*d*=difference between post-treatment and pre-treatment values.

**Table 3:** Percentage of cells staining for p53, Mdm-2 and p21 in primary and corresponding residual tumours after radiotherapy

	**p53**			**Mdm-2**			**p21**		
**Patient**	**Primary tumour %**	**Residual tumour %**	***d***	**Primary tumour %**	**Residual tumour %**	***d***	**Primary tumour %**	**Residual tumour %**	***d***
1	15	0	−15	44	88	44	8	0	−8
2	5	0	−5	100	100	0	66	14	−52
3	20	0	−20	100	100	0	32	0	−32
4	20	0	−20	79	100	21	18	8	−10
5	15	10	−5	100	0	−100	15	0	−15
6	0	0	0	85	100	15	0	17	17
7	30	60	30	100	100	0	10	0	−10
8	0	0	0	73	0	−73	0	11	11
9	0	0	0	27	0	−27	0	5	5
10	0	0	0	77	100	23	8	15	7
11	0	0	0	59	0	−59	32		
12	0	0	0	86	80	−6	42	10	−32
13	0	0	0	29	0	−29	0	0	0
14	0	0	0	13	77	64	29	5	−24
15	0	0	0	37	39	2	36	0	−36
16	8	10	2	100	100	0	34	13	−21
17	0	0	0	50	100	50	25	0	−25
18	0	0	0	34	59	25	0		
19	10	5	−5	92	100	8	30	24	−6
20	33	80	47	100	100	0	42	65	23
21	0	0	0	0	0	0	61	0	−61
22	6	0	−6	51	100	49	35	0	−35
Mean d			0.14			0.32			−15.2
s.d.			14.2			39.8			22.4

*d*=difference between post-treatment and pre-treatment values.

## References

[bib1] Allalunis-Turner MJ, Zia PK, Barron GM, Mirzayans R, Day III RS (1995) Radiation-induced DNA damage and repair in cells of a radiosensitive human malignant glioma cell line. Radiat Res 144: 288–293 7494872

[bib2] Bernstein C, Bernstein H, Payne CM, Garewal H (2002) DNA repair/pro-apoptotic dual-role proteins in five major DNA repair pathways: fail-safe protection against carcinogenesis. Mutat Res 511: 145–178 12052432 10.1016/s1383-5742(02)00009-1

[bib3] Beskow C, Agren-Cronqvist AK, Granath F, Frankendal B, Lewensohn R (2002) Pathologic complete remission after preoperative intracavitary radiotherapy of cervical cancer stage Ib and IIa is a strong prognostic factor for long-term survival: analysis of the Radiumhemmet data 1989–1991. Int J Gynecol Cancer 12: 158–170 11975675 10.1046/j.1525-1438.2002.01089.x

[bib4] Beskow C, Kanter L, Holgersson A, Nilsson B, Frankendal B, Avall-Lundqvist E, Lewensohn R (2006) Expression of DNA damage response proteins and complete remission after radiotherapy of stage IB-IIA of cervical cancer. Br J Cancer 94: 1683–1689 16685270 10.1038/sj.bjc.6603153 PMC2361310

[bib5] Dittmann K, Mayer C, Fehrenbacher B, Schaller M, Raju U, Milas L, Chen DJ, Kehlbach R, Rodemann HP (2005a) Radiation-induced epidermal growth factor receptor nuclear import is linked to activation of DNA-dependent protein kinase. J Biol Chem 280: 31182–31189 16000298 10.1074/jbc.M506591200

[bib6] Dittmann K, Mayer C, Rodemann HP (2005b) Inhibition of radiation-induced EGFR nuclear import by C225 (Cetuximab) suppresses DNA-PK activity. Radiother Oncol 76: 157–161 16024112 10.1016/j.radonc.2005.06.022

[bib7] Fuhrman CB, Kilgore J, LaCoursiere YD, Lee CM, Milash BA, Soisson AP, Zempolich KA (2008) Radiosensitization of cervical cancer cells via double-strand DNA break repair inhibition. Gynecol Oncol 110: 93–98 18589211 10.1016/j.ygyno.2007.08.073

[bib8] Fyles AW, Milosevic M, Wong R, Kavanagh MC, Pintilie M, Sun A, Chapman W, Levin W, Manchul L, Keane TJ, Hill RP (1998) Oxygenation predicts radiation response and survival in patients with cervix cancer. Radiother Oncol 48: 149–156 9783886 10.1016/s0167-8140(98)00044-9

[bib9] Haensgen G, Krause U, Becker A, Stadler P, Lautenschlaeger C, Wohlrab W, Rath FW, Molls M, Dunst J (2001) Tumor hypoxia, p53, and prognosis in cervical cancers. Int J Radiat Oncol Biol Phys 50: 865–872 11429213 10.1016/s0360-3016(01)01523-1

[bib10] Haupt Y, Maya R, Kazaz A, Oren M (1997) Mdm2 promotes the rapid degradation of p53. Nature 387: 296–299 9153395 10.1038/387296a0

[bib11] Hefferin ML, Tomkinson AE (2005) Mechanism of DNA double-strand break repair by non-homologous end joining. DNA Repair (Amst) 4: 639–648 15907771 10.1016/j.dnarep.2004.12.005

[bib12] ICRU (1985) International Comission on Radiation Units and Measurements: Dose and volume specifications for reporting intracavitary therapy in gynecology (Report 38). ICRU: Bethesda, MD, USA.

[bib13] Kastan MB, Onyekwere O, Sidransky D, Vogelstein B, Craig RW (1991) Participation of p53 protein in the cellular response to DNA damage. Cancer Res 51: 6304–6311 1933891

[bib14] Kim TJ, Lee JW, Song SY, Choi JJ, Choi CH, Kim BG, Lee JH, Bae DS (2006) Increased expression of pAKT is associated with radiation resistance in cervical cancer. Br J Cancer 94: 1678–1682 16721365 10.1038/sj.bjc.6603180 PMC2361323

[bib15] Kubbutat MH, Jones SN, Vousden KH (1997) Regulation of p53 stability by Mdm2. Nature 387: 299–303 9153396 10.1038/387299a0

[bib16] Lee CM, Shrieve DC, Zempolich KA, Lee RJ, Hammond E, Handrahan DL, Gaffney DK (2005) Correlation between human epidermal growth factor receptor family (EGFR, HER2, HER3, HER4), phosphorylated Akt (P-Akt), and clinical outcomes after radiation therapy in carcinoma of the cervix. Gynecol Oncol 99: 415–421 16157365 10.1016/j.ygyno.2005.05.045

[bib17] Lees-Miller SP, Godbout R, Chan DW, Weinfeld M, Day III RS, Barron GM, Allalunis-Turner J (1995) Absence of p350 subunit of DNA-activated protein kinase from a radiosensitive human cell line. Science 267: 1183–1185 7855602 10.1126/science.7855602

[bib18] Lees-Miller SP, Sakaguchi K, Ullrich SJ, Appella E, Anderson CW (1992) Human DNA-activated protein kinase phosphorylates serines 15 and 37 in the amino-terminal transactivation domain of human p53. Mol Cell Biol 12: 5041–5049 1406679 10.1128/mcb.12.11.5041-5049.1992 PMC360437

[bib19] Niibe Y, Nakano T, Ohno T, Tsujii H, Oka K (1999) Relationship between p21/WAF-1/CIP-1 and apoptosis in cervical cancer during radiation therapy. Int J Radiat Oncol Biol Phys 44: 297–303 10760422 10.1016/s0360-3016(99)00026-7

[bib20] Scheffner M, Werness BA, Huibregtse JM, Levine AJ, Howley PM (1990) The E6 oncoprotein encoded by human papillomavirus types 16 and 18 promotes the degradation of p53. Cell 63: 1129–1136 2175676 10.1016/0092-8674(90)90409-8

[bib21] Shintani S, Mihara M, Li C, Nakahara Y, Hino S, Nakashiro K, Hamakawa H (2003) Up-regulation of DNA-dependent protein kinase correlates with radiation resistance in oral squamous cell carcinoma. Cancer Sci 94: 894–900 14556663 10.1111/j.1349-7006.2003.tb01372.x PMC11160163

[bib22] Wilson CR, Davidson SE, Margison GP, Jackson SP, Hendry JH, West CM (2000) Expression of Ku70 correlates with survival in carcinoma of the cervix. Br J Cancer 83: 1702–1706 11104569 10.1054/bjoc.2000.1510 PMC2363444

